# Integrated hydrochar-bacteria strategy for heavy metal removal

**DOI:** 10.3389/fmicb.2026.1919518

**Published:** 2026-09-03

**Authors:** P. S. Karishma, Kavitha Sambasivam

**Affiliations:** 1 VIT School of Agricultural Innovations and Advanced Learning, Vellore Institute of Technology, Vellore, Tamil Nadu, India; 2 CO_2_ Research and Green Technologies Centre, Vellore Institute of Technology, Vellore, Tamil Nadu, India

**Keywords:** bacteria, co-remediation, heavy metal adsorption, heavy metal contamination, hydrochar

## Abstract

Heavy metal accumulation in soil and water is highly detrimental to environmental sustainability and living organisms because of its long-term retention, toxic nature, and potential to develop various health issues. Bacterial remediation strategy is considered relatively efficient, environmentally friendly and a sustainable approach with minimal adverse impacts when compared to other remediation methods. Bacteria exhibit strong heavy metal adsorption capability through various mechanisms including biosorption, bioaccumulation, bio-reduction and enzymatic conversion. However, bacterial agents are sensitive to various environmental stresses, which diminish their capability to accomplish a stable remediation outcome. Hydrochar, a carbonaceous material with higher porosity, surface area and abundant functional groups, can be integrated with bacteria as a supporting agent to overcome this challenge. The synergistic interaction of the hydrochar-bacterial system effectively eliminates heavy metals from environmental matrices by mechanisms such as complexation, ion-exchange, adsorption and redox reactions. Moreover, hydrochar can serve as a suitable habitat for bacterial colonization. Furthermore, hydrochar can be regarded as a cost-effective material, as it can be synthesized from various biowaste resources. This manuscript aims to elucidate the hazardous effects of heavy metals on living organisms, the mechanism of toxic metal removal by bacteria and hydrochar, and how the hydrochar-bacteria synergistic system helps to elevate the removal efficiency and bacterial viability through the supportive role of hydrochar. By discussing these interactions, this paper highlights the potential of hydrochar as a supporting matrix for bacteria, thereby enhancing the removal efficiency of hazardous metals. It further explores barriers and research frontiers potentially vital for making such systems scalable and environmentally benign in the long haul.

## Graphical Abstract

**Figure:**
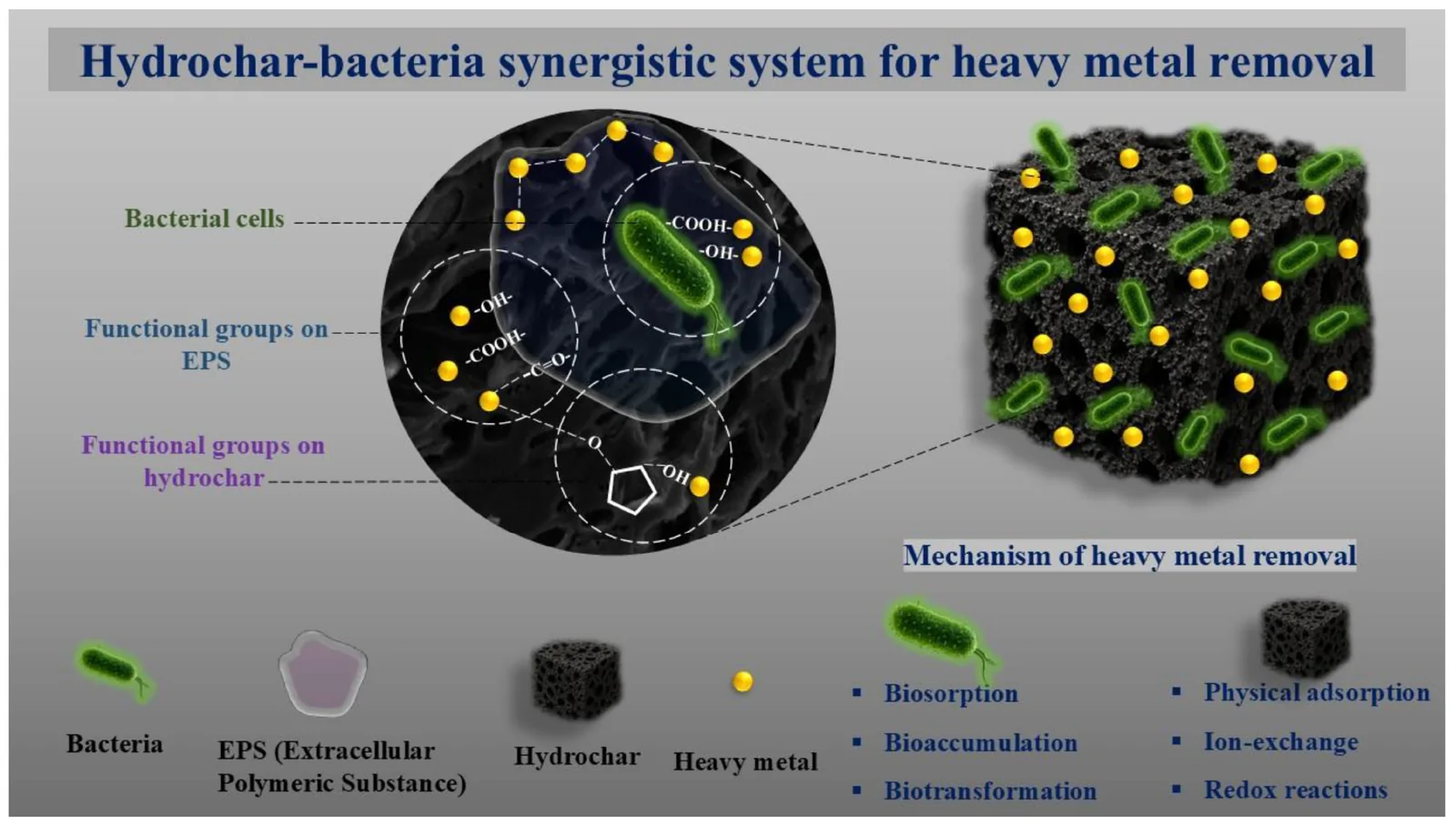

## 1 Introduction

Toxic metal ions are characterized by higher densities exceeding 5 g/cm^3^, including selenium (Se), lead (Pb), cadmium (Cd), arsenic (As), copper (Cu), mercury (Hg), nickel (Ni), chromium (Cr), and others. Due to their non-degradable and accumulative nature, they tend to persist in the environment for a protracted time and constitute serious environmental as well as health issues all over the globe (Ouyang et al., 2023). Anthropogenic activities are the chief contributors to heavy metal contamination.

The primary sources encompass industrial effluents, agricultural runoff, mining activities, untreated sewage, atmospheric deposition and stormwater runoff from urban areas (Oziegbe et al., 2025). The continuous discharge of these toxic elements into water bodies endangers humans and animals due to their cumulative bioaccumulation (Zulfiqar et al., 2019). The covalent bond formed by the metalloids constitutes the basis of their toxic effects. This allows them to covalently bind with organic compounds and form lipophilic compounds that can interact with non-metallic elements in cellular macromolecules, producing toxicity (Briffa et al., 2020). Heavy metal pollutant aggregation aggravates aquatic pollution and compromises drinking water quality, food production, and crop safety, ultimately jeopardising human health (Wang et al., 2022). Therefore, exploring mitigation methods and remediating heavy metal contamination is crucial. Several removal strategies are being explored currently, such as ion exchange, membrane filtration, chemical precipitation and coagulation-flocculation (Table 1). These approaches often exhibit poor selectivity, stripping necessary ions along with toxic metals and producing hazardous byproducts, such as sludge (Zamora-Ledezma et al., 2021). The ion-exchange technique shows high removal efficiency and heavy metal recovery, but it is found to be expensive and sensitive to pH and other competing ions (Razzak et al., 2022). In the coagulation and flocculation technique, the removal is rapid and easy to implement; on the other hand, the selectivity is low and produces toxic sludge (Sukmana et al., 2021). Due to operational simplicity, chemical precipitation is widely employed; however, it generates excessive sludge and might lead to elimination of necessary ions (Ayach et al., 2024). Though highly efficient in metal separation even at trace levels, membrane filtration is restricted by its high cost and frequent membrane fouling (Shofia et al., 2025).

**TABLE 1:** Advantages and drawbacks of conventional remediation methods.

Remediation method	Advantages	Drawbacks	References
Chemical precipitation	Simple, cost effective, widely used	Generates large volumes of sludge, may remove essential ions	Malik et al., 2017
Coagulation-flocculation	Rapid, easy to implement, effective for suspended solids	Low selectivity, produces toxic sludge	Qasem et al., 2021
Ion exchange	High removal efficiency, can recover metals	Expensive, sensitive to pH and competing ions	Kowalik-Klimczak, 2025
Membrane based filtration	Excellent separation efficiency, effective for low metal concentrations	Elevated energy cost, membrane fouling, expensive	El Batouti et al., 2021

Bioremediation has been recognized as a sustainable, environmentally compatible and economically feasible alternative to circumvent the limitations of conventional physico-chemical remediation methods for heavy metal elimination (Das et al., 2025). In bioremediation, most predominantly employed agents are microorganisms (Table 2). Microbial bioremediation processes have been proven as a resource-efficient approach in the purging of heavy metals from contaminated air and water sources, wherein pollutants are transformed into non-hazardous end products (Choudhury and Chatterjee, 2022). Microorganisms are instrumental in soil restoration, and opting for an adaptive strain is pivotal. Proteobacteria exhibit tolerance to lead and zinc, whereas *Bacteroidetes* and *Firmicutes* are ideal for arsenic-polluted sites. The bioavailability and toxicity of heavy metals ions are determined by their oxidative states, and microorganisms can modify the state via enzymatic transformation (Imran et al., 2025).

**TABLE 2:** Removal efficiency of bacterial species on various heavy metals.

Bacterial species	Heavy metal	Removal efficiency	References
*Bacillus wiedmannii*.	Chromium	70.27%	Adhikary et al., 2025
*Staphylococcus devriesei*	Chromium Cadmium	98% 81.2%	Shabaan et al., 2025
*Bacillus cereus*	Lead Cadmium Chromium	69% 54% 43%	Ayangbenro and Babalola, 2020
*Pasteurella aerogenes* aUoR-24	Copper Zinc Nickel	78.62% 90.72% 70.43%	Qurbani et al., 2025
Zinc resistant XZN4 strain	Zinc	92.8%	Huang et al., 2020
*Pteridium aquilinum*	Antimony Lead Mercury Zinc Arsenic	66.08% 22.99% 15.15% 14.83% 7.39%	Chambi Mamani et al., 2025
*Serratia marcescens*	Chromium	>80%	Xu et al., 2018
*Comamonas sp.* HMZC	Zinc Nickel Cadmium	>98%	Rajasekar et al., 2025
*Pseudomonas aeruginosa*	Copper Cadmium Lead	82.35% 50% 91.66%	Shah et al., 2025
Sulfate-reducing bacteria	Lead Zinc	88.3% 98.7%	Yan et al., 2023
*Serratia marcescens* QZB-1	Manganese	98.4%	Huang et al., 2023
*T. longibrachiatum*	Chromium Zinc Lead	87% 67% 48%	Firincã et al., 2024
*Bacillus megaterium*	Lead	73%	Njoku et al., 2020
*Bacillus xiamenenensis*	Lead	97%	Mohapatra et al., 2019

The fundamental concept of microbial remediation resides in enzymatic transformation of pollutants. Microbial remediation is anchored by enzymes such as oxidase, oxido-reductase, methyltransferase, and also through metabolic activities (Medfu Tarekegn et al., 2020). Moreover, numerous functional groups located on the bacterial cell surface play a vital role in adhesion of heavy metals, either to the cell membrane or via intracellular reduction (Zhou et al., 2023). A microbial approach for the removal of heavy metals is considered a promising tool. Nevertheless, it has noticeable challenges in separating and reusing the cells and instability of microbes under extreme environmental conditions such as temperature, pH and high concentration of heavy metals (Wu et al., 2021).

Microbial immobilization escalates the efficiency of microbial remediation, and selecting a compatible immobilizing carrier is crucial. As a bio-augmentation method, microbial immobilization involves embedding microorganisms on a host material by means of a physical or chemical method (Bouabidi et al., 2019). Materials such as activated carbon, carbon nanotubes, biochar, hydrochar, graphene and graphene derivatives are commonly employed for this purpose. Integration of hydrochar as a support matrix for microbial immobilization is emerging as a groundbreaking methodology for heavy metal remediation. Hydrochar, as a carbon-rich material produced from biomass under hydrothermal conditions, confers multiple advantages. Convenient synthesis process, availability of diverse feedstock, low operational cost and surface functional group abundance make it a potential candidate for pollutant adsorption applications (Wu et al., 2025). Hydrochar has achieved widespread recognition among the scientific community due to its eco-friendly and resource-efficient nature, allowing it to become a highly efficient and sustainable adsorbent for the remediation of pollutants from aqueous environments (Nadarajah et al., 2021). Despite the theoretical and practical potential, hydrochar-assisted bacterial remediation research in this field remains at a very early stage. Most existing studies have investigated hydrochar-based adsorption and bacterial remediation as separate approaches, whereas direct investigation of hydrochar-immobilized bacteria for heavy metal removal is extremely limited. Therefore, this review critically examines the hydrochar-assisted bacterial remediation of toxic metals as an emerging hybrid strategy. It discusses heavy metal toxicity, bacterial remediation mechanisms, hydrochar properties, immobilization strategies, possible synergistic mechanisms between hydrochar and bacteria, current research gaps, future directions required for laboratory validation, scale-up and field validation.

### Objective of the study

1. To critically examine the synergistic effect of bacteria and Hydrochar for enhanced heavy metal removal, with emphasis on adsorption, biosorption, bioaccumulation, and biotransformation mechanisms.
2. To evaluate the role of hydrochar as a supporting matrix in improving bacterial survival, immobilization, stability, and remediation efficiency under heavy metal stress conditions.
3. To compare the remediation performance of bacteria alone, hydrochar alone, and integrated hydrochar-bacteria systems for contaminated water and soil treatment.
4. To identify current research gaps, practical challenges, and prospects for developing sustainable, low-cost, and large-scale hydrochar-bacteria remediation technologies.
5. To the best of our knowledge, dedicated review articles focusing specifically on this topic remain very limited. Accordingly, this review seeks to bridge this knowledge gap by critically evaluating the available literature and identifying key opportunities for future investigation.
6. To compile and critically analyse scattered studies on hydrochar-bacteria interactions, adsorption behavior, and microbial-assisted metal removal mechanisms in a single platform.
7. To highlight the distinct advantages of hydrochar over commonly reviewed Biochar, particularly its suitability for wet biomass feedstocks and unique surface chemistry.
8. To identify existing research gaps and propose future directions for translating hydrochar-bacteria systems from laboratory studies to practical large-scale applications.

## 2 Heavy metal pollution

**FIGURE 1:**
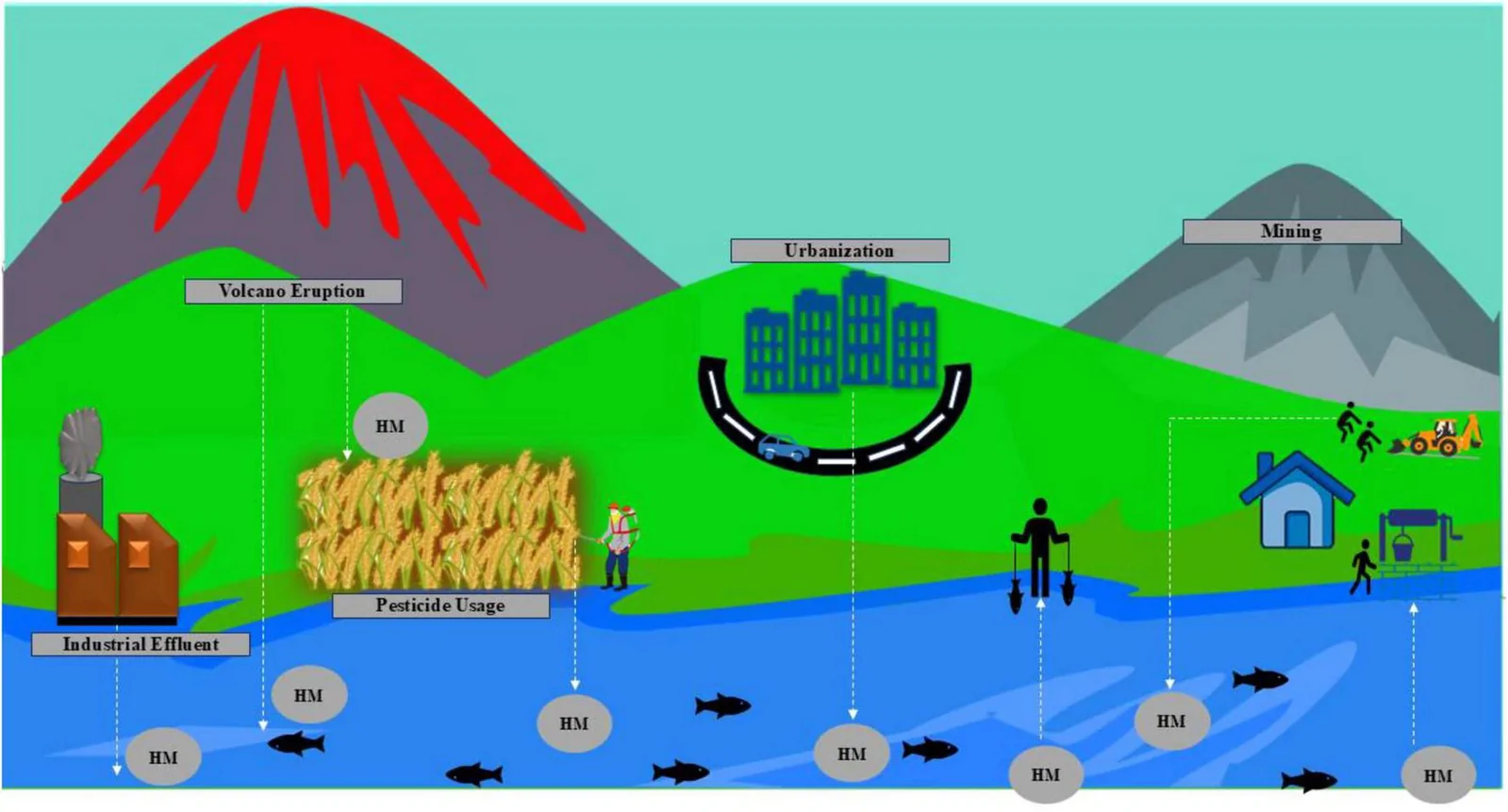
Heavy metal pollution sources.

Heavy metals are generally defined as elements having atomic number greater than 20 and an elemental density more than 5 g/cm^3^(Ali and Khan, 2018). To support any physiological and biochemical functions, these metals are crucial in living organisms in minimal concentrations. (Jaishankar et al., 2014). These metals are categorized as essential and non-essential based on their involvement in biological systems. Essential metals such as Zn, Mn, Cu and Fe are vital for living organisms in trace levels. Cd, Cr, Pb and Hg belong to non-essential metal ions which lack established biological function (Ali et al., 2019). Heavy metal pollution in the environment originates primarily from anthropogenic activities such as industrialization, mining, smelting, urbanization, and agricultural practices, alongside natural sources including volcanic eruptions, weathering, and rock abrasion (Figure 1). Wastewaters from industries, mining waste, landfill leachates, municipal effluents, and urban runoff are significant contributors to heavy metal pollution in terrestrial and aquatic ecosystems (Hama Aziz et al., 2023). Soils around smelting sites, industrial regions, and urban areas are often heavily contaminated by metals such as lead (Pb), cadmium (Cd), arsenic (As), mercury (Hg), chromium (Cr), copper (Cu), and zinc (Zn) due to direct discharges and atmospheric deposition (Parvez et al., 2023). These metals undergo numerous environmental processes when entered into environment and disseminate in different environmental matrices such as water, soil and atmosphere (Sukhjadeorao Dongre, 2021). The fate of the metals depends on the quantity and degradation capacity (Laoye et al., 2025). Chlorine and caustic soda production industries are the major contributors to the release of mercury into the environment, while cadmium concentration is higher in rocks, mineral fertilizers, soils and coal (Zaynab et al., 2022). In copper slags, Cobalt can be seen in significant amount and this metal is listed among critical minerals by several international agencies (Adnan et al., 2024).

## 3 Biological impact of heavy metals

The environmental and health risk of heavy metal ions are profound. Heavy metal ions are persistent, toxic, and bioaccumulative, posing risks to ecosystems and human health even at low exposure levels. Toxic effects include damage to neurological, hepatic, renal, pulmonary, reproductive, dermal, and gastrointestinal systems, as well as carcinogenic potential (Al-Tamimi et al., 2025). For instance, heavy metal tainting in water sources has been strongly associated with increased risks of stomach cancer and other serious health conditions (S. Chen and Ding, 2023). In agricultural soils, heavy metals impact the growth and development of cereal crops by inducing physiological stress, chlorosis, and stunted growth, thereby threatening food security and sustainability (Vasilachi et al., 2023). The biological accumulation of heavy metals in medicinal plants and food chains further magnifies human exposure risks, underscoring public health concerns (Hlihor et al., 2022). Heavy metals can gain entry into the body through several routes, including consumption of food and water, inhalation of polluted air, and absorption through skin and adversely affect biological processes (Tchounwou et al., 2012). Exposure to heavy metals has been implicated in diverse adverse health outcomes. The pathway of heavy metals into the human body often involves the food chain. Plants grown in contaminated soils absorb heavy metals, which bioaccumulate and bio magnify as they move through trophic levels. Consumption of food plants contaminated with heavy metals can lead to nutrient deficiencies due to altered plant metabolism and may directly impart toxic metals to humans. This is of particular concern in developing countries reliant on local agriculture where heavy metal contamination is prevalent (Khan et al., 2015).

### 3.1 Chromium

Hexavalent chromium is widely used in many industrial processes, such as steel welding and electroplating. These processes generate wastewater, particularly contaminated wastewater, which pollutes the environment and poses a major threat to human health. (Iyer et al., 2023). It is acknowledged that chromium is a harmful, mutagenic, and carcinogenic metal that poses a significant risk to the population. Trivalent chromium and hexavalent chromium are the two stable oxidation states that are most prevalent in the environment. While hexavalent chromium is highly soluble, more toxic, and a strong carcinogen, trivalent chromium is typically linked to less toxicity (Khan et al., 2024). According to a study done on both male and female populations, the lifetime prevalence of Cr (VI) exposure among men was 23% in controls and 30% in cases. 24% of cases and 19% of controls had nickel exposure, and 77.7% of cases and 83% of controls also had co-exposure to Cr (VI). Women, on the other hand, had significantly lower exposure levels; in both cases and controls, Cr (VI) exposure was recorded at 5%. In both groups, the percentage of females exposed to nickel was 3%. The study also showed that prolonged exposure to both Cr (VI) and nickel was associated with similar increases in lung cancer odds ratios for both sexes (Behrens et al., 2023). Nasal septum perforation (chromium ulcers), acute and chronic upper-airway irritation and rhinitis, asthma and other chronic lung diseases (such as pulmonary fibrosis and COPD), allergic contact dermatitis, and skin ulcers can all result from occupational Cr (VI) exposure (Zhang et al., 2023).

Chromium is primarily taken up by plant roots through the process of root uptake and thus is sequestered in plant roots, which also represent the main area of plant chromium uptake from the soil. Additionally, chromium is one of the least migratory heavy metals in the root system of plants (Shukla et al., 2007). Its concentration in roots is reported to be significantly higher than in shoots, primarily due to its limited mobility within the plant (Gupta and Sinha, 2006). Chromium usually has three significant harmful effects on plants. The first is that it impedes the production of vital pigments necessary for photosynthesis. This includes the inhibition of the production of anthocyanins and other essential pigments used during the process of photosynthesis. Secondly, chromium causes an increase in the number of other phytochemicals produced by plants (e.g., glutathione, ascorbic acid), which can negatively impact physiological functions. Finally, chromium causes an increase in the production of alternative metabolites (e.g., histidine, phytochelatin), which assist in providing the plant with the ability to handle stress (Ghori et al., 2019). The initial location at which plant roots interact physically with soil is called the root-soil interface. The degree to which any particular plant species is capable of absorbing chromium into tissues will vary based upon both the type(s) of plant being examined and the specific chemical/ionic form(s) in which chromium exists in the environment (e.g., Cr (III) versus Cr (VI)). Additionally, the formation of chromium-complexes with organic ligands will increase chromium-bioavailability and uptake by plants (Zulfiqar et al., 2023). Chromium has been reported to significantly inhibit seed germination in a variety of plant species, including vegetables such as cauliflower (*Brassica oleracea* L.) and *Citrullus vulgaris*, as well as major crops like wheat (*Triticum aestivum* L.), barley (*Hordeum vulgare* L.), and maize (*Zea mays* L.) (Ao et al., 2022). In a representative transcriptomic study on rice plants exposed to chromium (VI) ions, researchers discovered major changes to the overall transcriptional profile of the plant’s genome after exposure to the toxic metal. In general, genes associated with membrane transport, signal transduction (i.e., how information is communicated between different systems), and xenobiotic metabolism (i.e., how the body functions with respect to foreign substances) were all upregulated by chromium (VI) exposure; whereas, genes related to cell division, and energy production were downregulated by chromium (VI) (Ali et al., 2023). It has been reported that *Vigna radiata* plants cultivated in soil contaminated with 50% chromium exhibited a significant reduction in amino acid, starch, and chlorophyll contents (Abdullah et al., 2023).

### 3.2 Cadmium

The metal cadmium was identified for the first time in 1817 bystromeyer during an examination of impurities in zinc. The natural form of cadmium ranges from pure cadmium (Cd^0^) to various cadmium compounds, including cadmium carbonate, chloride, oxide, sulfate and sulfide. Cadmium can be found throughout the earth’s crust and is released into the environment through natural processes (rock weathering, volcanic eruptions) in both land and ocean environments. However, it should also be noted that human activities (the production of cadmium) contribute significantly to cadmium emissions; for example, zinc production has an average Zn/Cd ratio of 200:1 to 400:1, so zinc production can be considered a significant source of cadmium emissions (Rasin et al., 2025). Cadmium (Cd) has a pronounced ability to accumulate and remain in soils where it eventually moves into the plant’s metabolic system. As cadmium builds up in the consumable tissue of plants such as fruit and seed, it can be transferred through the food chain. In humans, the kidneys are the organs most vulnerable to cadmium toxicity; about 30% of the total cadmium burden in the body resides in the renal tubular areas (Peana et al., 2023). The human body retains cadmium (Cd) for an extended period of time, with reported half-lives of 16 to 30 years. Exposure to low doses of Cd over a long period has been linked to the emergence of several respiratory diseases including emphysema, asthma, and chronic bronchitis, and can also lead to high blood pressure. Long-term exposure to cadmium may lead to critical health problems such as cancer, leukemia, and genotoxicity (Charkiewicz et al., 2023). Long-term exposure to inhaled cadmium has been linked to respiratory diseases like emphysema and chronic inflammation of the nose, pharynx, and larynx as well as a reduction in the ability to smell (olfaction) (Nordberg et al., 2018). As with many other metals that have been shown to cause toxicity and cancer by causing oxidative stress, cadmium does not have redox activity; however, it increases the formation of reactive oxygen species (ROS within cells) resulting in oxidative damage to lipids, proteins, and DNA (Ðukić-Ćosić et al., 2020). The kidney functions as the most important organ for any kind of chronic dietary exposure. Nephrotoxicity from cadmium is caused by the impaired ability of the proximal tubule to reabsorb (Järup and Akesson, 2009). In addition, cadmium can disrupt female reproductive hormone production and causes the ovaries to have hemorrhagic alterations and induces vascular endothelial injury, which also affect the biosynthesis of progesterone. Prolonged cadmium exposure may lead to reduced bone mass and lower bone mineral density; it can also greatly increase an individual’s risk of fractures as well as produce skeletal lesions. Furthermore, cadmium disturbs the metabolism of important minerals including magnesium, zinc, calcium, iron, and copper, reduces normal vitamin D metabolism as well as decreases phosphate uptake in bone (Suhani et al., 2021). Visible occurrences of cadmium toxicity in plants generally include stunted growth and chlorosis. When cadmium levels are elevated, plant development will be greatly decreased. Various physiological processes are negatively affected by the presence of cadmium, including the impairment of carbon fixation and lowering of chlorophyll content, both of which result in reduced photosynthetic activity (Haider et al., 2021).

## 4 Hydrochar production

Hydrochar, a solid material with abundant carbon content produced by hydrothermal carbonization (HTC). HTC is a thermochemical process by which wet biomass is dissolved in hot, pressured water, usually 180–260 °C under autogenous pressure for short periods of time (minutes to hours). HTC employs complex chemical reactions including dehydration, decarboxylation, polymerization and aromatization, transforming lignocellulosic biomass into hydrochar with higher carbon content and energy density than the raw feedstock (Pasipanodya et al., 2025). The properties of the resulting products are primarily governed by key operational parameters, including pressure, water-to-biomass ratio, residence time, and the reaction temperature. During hydrothermal carbonization (HTC), hydrochar is the desired solid product, with a typical yield of about 40-70%wt (Zhang et al., 2019). During the hydrolysis reaction water has been observed to act as both catalyst and reactant. An increase in temperature decreases the dielectric constant of water making it a more effective medium for dissolution and reaction of organic compounds (Sharma R. et al., 2020). The hydrolysis of biomass is initiated by hydronium (H_3_O^+^) ions formed through the self-ionization of water under hydrothermal conditions. As the temperature rises, the ionization constant of water increases, enhancing its catalytic role in the hydrolysis of organic (Yang et al., 2018). The carbonization process is usually followed by a reduction in pH of biomass and process water due to the formation of organic acids such as levulinic acid, lactic acid and acetic acid. These organic acids help to hydrolyze and decompose Small-chain polymers and monomers to broken down into significantly smaller molecular fragments. (Sharma H. B. et al., 2020). Hydrochar generally exhibits a porous structure characterized by micropores and abundant oxygen-containing functional groups; however, it typically shows a lower degree of aromatic carbonization and reduced stability compared with biochar produced through high-temperature pyrolysis (Holliday et al., 2022). Hydrochar has its advantages and drawbacks apart from biochar and activated carbon (AC). Biochar with slow pyrolysis has an increased fixed carbon content, more aromaticity and a more stable carbon structure that is helpful for soil amendment and carbon sequestration. Activated carbon is a high-density, porous surface product readily activated from biochar or other precursors by chemical/physical activation that absorbs both organic and inorganic pollutants. Hydrochar typically exhibits a lower surface area and a less pore complexity structure than activated carbon; however, it contains abundant surface functional groups that enhance its adsorption capacity and catalytic potential. Moreover, hydrothermal carbonization is suitable for wet biomass feedstock without needing to be dried prior, can be more compact and has a simpler reactor architecture, and can retain certain nutrients in hydrochar that are beneficial for selective applications (Gale et al., 2021).

Hydrochar production depends greatly on the biomass feedstock type as it relates to yield, physicochemical properties, and potential applications. A study by demonstrated that feedstock composition has a greater influence on hydrochar quality than hydrochar yield alone. Although whitewood and rapeseed produced comparable hydrochar yields, rapeseed exhibited a higher heating value (HHV), likely due to its greater carbon-rich constituents and enhanced carbon retention during hydrothermal carbonization. In contrast, *Laminaria digitata* generated hydrochar with a relatively high yield but poor fuel quality because its high inorganic mineral content resulted in elevated ash content and reduced energy density. These findings indicate that hydrochar yield should not be used as the sole criterion for evaluating feedstock suitability; instead, elemental composition, ash content, and carbon enrichment should be considered to accurately assess its potential applications (Güleç et al., 2022). The comparison between hydrochar and pyrochar demonstrates that a higher specific surface area does not necessarily translate into superior adsorption performance. Although pyrochar possessed greater porosity and surface area, the abundance of oxygen-containing functional groups on microalgal hydrochar enhanced its affinity for heavy metals, indicating that surface functionalization can outweigh physical characteristics in adsorption processes. In addition, the direct processing of wet microalgal biomass by HTC eliminates the need for drying, reducing energy consumption and improving the overall feasibility of hydrochar production for large-scale environmental applications (Park and Kim, 2023). Integrating ultrasound with hydrothermal carbonization enhances biomass conversion through acoustic cavitation, where the collapse of microbubbles generates localized high temperatures, pressures, and microjets that disrupt the lignocellulosic matrix. This structural disruption increases the accessibility of cellulose and hemicellulose to hydrolytic reactions, while improved heat and mass transfer accelerates hydrolysis, dehydration, decarboxylation, and subsequent condensation/polymerization reactions. Consequently, greater carbon retention and secondary carbon deposition within the solid phase enhance hydrochar yield and carbonization efficiency under optimized conditions. Simultaneously, the release of volatile compounds and cavitation-induced microfractures facilitate pore development, whereas the relatively mild HTC conditions preserve abundant oxygen-containing functional groups (e.g., hydroxyl, carboxyl, and carbonyl groups) (Madusari et al., 2023). Physicochemical properties of corn stalk hydrochar are strongly influenced by HTC process severity. Increasing the reaction temperature intensified hydrolysis, dehydration, and decarboxylation reactions, promoting the decomposition of cellulose and hemicellulose while enriching the carbonaceous matrix. The progressive removal of volatile compounds and degradation of structural polysaccharides generated additional voids within the hydrochar, contributing to increased pore development. Simultaneously, the dissolution of alkali and alkaline earth metals into the aqueous phase facilitated demineralization, whereas lignin-derived intermediates underwent condensation and aromatization, forming a more stable carbon framework. However, excessively severe HTC conditions accelerated the decomposition of organic matter into liquid products, reducing hydrochar yield despite increasing the degree of carbonization (Yang et al., 2021).

## 5 Hydrochar properties

### 5.1 Surface porosity

Hydrochar has a relatively large surface area but is smaller than activated carbon and high-temperature biochar. Hydrothermal carbonization produces hydrochar with pore structures following polymerization and dehydration reactions. These pores are adsorbent sites. Post-treatment or chemical activation can augment the surface area. The porous matrix contains micro- and mesopores, a balance that is beneficial for the attachment of both small and large molecules (Ferrentino et al., 2020; Naranjo et al., 2023). Demonstrated that the development of surface area in rice husk hydrochar occurs through a sequential mechanism involving hydrothermal carbonization followed by alkali activation. During HTC, the hydrolysis of cellulose and hemicellulose removes labile biomass components, generating initial voids, while lignin-derived condensation reactions stabilize the carbon framework. Subsequent NaOH activation selectively etches poorly carbonized regions, removes pore-blocking organic residues, and opens previously inaccessible micropores, thereby increasing the BET surface area and pore accessibility. However, the study further revealed that the enhanced surface area did not significantly increase the equilibrium adsorption capacity, indicating that adsorption performance depends not only on pore development but also on the abundance and accessibility of surface oxygen-containing functional groups. Porosity of glucose-derived hydrochar evolves through the synergistic effects of hydrothermal carbonization and subsequent KOH activation. During HTC, glucose undergoes dehydration to form 5-hydroxymethylfurfural (HMF), followed by polycondensation and aromatization reactions that generate oxygen-rich carbon microspheres with a partially developed carbon framework. Although these hydrochars contain abundant oxygen-containing functional groups that serve as reactive sites for activation, their intrinsic porosity remains limited because many pores are closed or occupied by condensed carbonaceous species. Subsequent KOH activation selectively gasifies the carbon matrix, while metallic potassium intercalates into and expands the aromatic carbon layers, creating new micropores and widening existing ones into interconnected mesopores. The combined effects of carbon etching, lattice expansion, and removal of activation products substantially increase pore volume and specific surface area, demonstrating that the exceptional porosity of hydrochar-derived activated carbon originates primarily from chemical activation rather than hydrothermal carbonization alone (Lin et al., 2023). Pore development in sugarcane bagasse-derived hydrochar occurs through a two-stage mechanism involving hydrothermal carbonization followed by steam activation. During HTC, the hydrolysis of cellulose and hemicellulose, together with dehydration and decarboxylation reactions, generates an initial carbon framework containing closed pores and structural defects, while lignin-derived condensation reactions stabilize the carbon matrix. Subsequent steam activation selectively gasifies the less ordered carbon domains, removes pore-blocking carbonaceous deposits, and creates interconnected micropores by etching the carbon framework. Continued gasification widens adjacent micropores into mesopores through pore-wall erosion, increasing pore volume and accessibility. However, excessive activation can over-gasify the carbon matrix, leading to pore-wall collapse and reduced structural integrity, indicating that pore development is governed by a balance between pore generation and carbon consumption rather than activation severity alone (Congsomjit and Areeprasert, 2021). In a study with shells from *Camellia oleifera*, the HTC temperature increased from 150 °C to 300 °C, resulting in rougher surfaces with more visible pore structures that indicate higher temperatures accelerate surface development and pore formation (Chen et al., 2021).

Hydrochar pore size, typically are less than 10 nm, with the presence of micropores (2 nm) and mesopores (2–50 nm) which are necessary for effective adsorption capabilities. The increase in temperature triggers dehydration, decarboxylation, and removal of volatile matter which creates and expands these pores, increasing the total pore volume and BET surface area (Arauzo et al., 2020). In HTC, the ambient temperature must be controlled effectively as too high temperatures can cause structural changes or pore collapse with negligible surface area and volume (Satira et al., 2021).

### 5.2 Functional group

Hydrochar surfaces have an abundance of oxygen-containing functional groups such as hydroxyl, carboxyl, and carbonyl moieties that arise from incomplete carbonization in relatively low-temperature, aqueous HTC conditions. These polar functions play a critical role in adsorption processes through their capacity to promote hydrogen bonding, surface complexation and ion-exchange interactions. Oxygen-containing groups decrease as HTC temperature increases through dehydration, decarboxylation and condensation reactions, while carbon content and aromaticity increase to modify the adsorption behavior of the material. Thus, surface functional groups are highly correlated with feedstock properties and process parameters (Zhu et al., 2024). Hydrochar’s oxygen-rich surface chemistry is also highly reactive with heavy metal ions, making it well suited for water treatment. In morphological terms, hydrochar is generally characterized by a core-shell interaction with particle size ranges from 2 to 10 m, and the hydrophobic core contains relatively stable oxygen functions such as ether, pyrone, and quinone groups, while the hydrophilic shell contains more reactive oxygenated groups, such as carbonyl and hydroxyl/phenolic moieties. Unlike biochar, it has typically no known morphology (Sharma H. B. et al., 2020).

Surface chemistry of hydrochar is governed by the dynamic transformation rather than the simple removal of oxygen-containing functional groups during hydrothermal carbonization. Hydrolysis of cellulose and hemicellulose exposes reactive carbon sites, while dehydration and decarboxylation selectively eliminate unstable hydroxyl and carboxyl moieties, increasing the degree of carbonization. Simultaneously, lignin-derived intermediates undergo condensation and aromatization to form a stable aromatic carbon framework, within which oxygen-containing functional groups such as hydroxyl, carbonyl, carboxyl, and ether groups remain predominantly at the edges of the carbon structure. These observations indicate that HTC generates hydrochars with chemically heterogeneous surfaces, where the balance between deoxygenation and the retention of reactive oxygen functionalities is dictated by the extent of carbonization rather than by complete functional group (Başakçılardan Kabakcı and Baran, 2019). Cai et al. (2016) employed FTIR spectroscopy to demonstrate that the surface chemistry of tobacco stalk hydrochar undergoes systematic transformation during hydrothermal carbonization. The progressive weakening of the broad O–H stretching band (3200–3600 cm^–1^) indicated the consumption of hydroxyl groups through dehydration, while the reduced intensity of aliphatic C–H stretching bands (2920–2850 cm^–1^) reflected the decomposition of aliphatic carbon chains and their conversion into more condensed aromatic structures. Simultaneously, the decline in the carbonyl absorption band (1700–1735 cm^–1^) suggested that oxygen-containing intermediates generated during hydrolysis were subsequently removed through decarboxylation and decarbonylation reactions. The marked reduction of C–O–C and C–O vibrations within the 1000–1160 cm^–1^ region further confirmed the extensive hydrolysis of cellulose and hemicellulose, resulting in the cleavage of glycosidic linkages and dissolution of carbohydrate-derived products. Superior adsorption performance of hydrochar primarily arises from the retention of oxygen-containing functional groups during hydrothermal carbonization rather than from extensive pore development alone. These oxygenated functionalities provide chemically active sites for hydrogen bonding, ion exchange, and surface complexation, while partially condensed aromatic domains contribute to π–π interactions. Consequently, hydrochar possesses a multifunctional surface capable of adsorbing a broad range of contaminants through multiple simultaneous mechanisms. Unlike pyrochar and activated carbon, whose adsorption is governed predominantly by aromatic interactions, hydrochar exhibits enhanced adsorption selectivity due to its higher density of oxygenated surface functionalities, indicating that surface chemistry is often a more important determinant of adsorption performance than specific surface area alone (Vu et al., 2025).

### 5.3 Elemental composition

Hydrochar contains a characteristic elemental composition predominantly composed of carbon (45–80 wt%), hydrogen (2–6 wt%), oxygen (10–45 wt%) and nitrogen (0.2–5 wt%) that is slowly synthesized during hydrothermal carbonization (HTC) of wet biomass. In HTC, dehydration, decarboxylation, demethylation and polymerization induce the release of volatile components and the progressive carbon enrichment of the solid phase (Kambo and Dutta, 2015). As HTC temperatures rise to 300–350 °C, carbon content typically increases from 50–75 wt% to >40–50 wt% and oxygen and hydrogen fall from 15–30 wt% to 3–5 wt%, respectively. These changes result in a decrease in the O/C (0.2–0.5) and H/C (0.5–1.0) atomic ratios in both batch and continuous HTC, indicating a greater degree of carbonization and aromaticity. Nitrogen in hydrochar is typically retained at ∼1–4 wt% and is mainly present as pyrrolic and pyridinic species, whereas ash content increases to ∼5–20 wt% due to the concentration of inorganic constituents (e.g., Ca, K, and P) in the solid phase (Rivas-Arrieta et al., 2024). Hydrochar preserve between 1 and 3 wt% nitrogen in protein-rich feedstocks such as algal biomass as surface amines or heterocyclic groups that increase heavy metal chelation in single and mixed contaminants (González-Fernández et al., 2024). Decoupled hydrothermal carbonization of cellulose produces hydrochar spheres with negligible nitrogen content, reflecting the intrinsically low nitrogen level of the feedstock (∼0.1–0.3 wt%). Although elevated pressures (30–100 bar) enhance cellulose hydrolysis, they do not promote nitrogen incorporation, resulting in hydrochar with nitrogen contents of approximately 0.05–0.1 wt%; XPS analysis typically detects less than 1% pyridinic nitrogen. Under these conditions, the process favors carbon sphere formation rather than heteroatom doping (Yu et al., 2022). Hydrochar retained substantial amounts of hydrogen and oxygen-containing functional groups due to the relatively mild hydrothermal carbonization conditions, resulting in a material with limited aromatic condensation and high chemical reactivity. This composition favored the presence of labile dissolved organic matter and partially carbonized aliphatic structures, making the hydrochar more biodegradable than pyrolysis-derived biochar. Consequently, although these oxygenated functional groups improve hydrophilicity and nutrient interactions, the relatively high H/C and O/C characteristics reduce long-term carbon stability and can stimulate excessive microbial respiration, leading to phytotoxic effects at high application rates (Bona et al., 2023). Waste paper sludge-derived hydrochar is typically characterized by a relatively high carbon content and reduced hydrogen and oxygen fractions compared with the parent feedstock, reflecting extensive dehydration and decarboxylation reactions during hydrothermal carbonization. The progressive decrease in the H/C and O/C atomic ratios indicates enhanced carbon aromatization, increased structural condensation, and the formation of a more stable carbonaceous matrix with improved physicochemical stability. Simultaneously, the inorganic mineral fraction inherited from the sludge, particularly calcium- and silica-rich ash, contributes significantly to the hydrochar’s elemental composition and can influence its thermal behavior, alkalinity, and adsorption performance (Tu et al., 2021). Hydrochar synthesized from avocado seed at 200 °C for 2 h exhibited a marked increase in carbon and nitrogen contents, accompanied by a decrease in hydrogen and oxygen contents relative to the raw biomass, confirming that hydrothermal carbonization promoted extensive dehydration, decarboxylation, and aromatization reactions. These elemental transformations lowered the H/C and O/C atomic ratios, indicating the development of a more condensed and chemically stable aromatic carbon framework with enhanced resistance to thermal degradation. Loss of oxygen-rich volatile compounds concentrates the carbon matrix and facilitates the formation of conjugated aromatic domains, whereas the retained oxygen- and nitrogen-containing functional groups maintain sufficient surface polarity for electrostatic interactions and anion exchange (Durán-Plazas et al., 2025).

## 6 Hydrochar mechanism on heavy metal adsorption

### 6.1 Physisorption

Hydrochar promotes heavy-metal removal predominantly through physisorption, governed by non-covalent interactions such as van der Waals forces, electrostatic attractions, and pore-diffusion-driven entrapment, rather than through specific chemical bonding. This behavior is largely attributed to its mesoporous architecture and surface charge characteristics. Mesopores in the range of 2–50 nm, with a specific surface area typically between 50 and 300 m^2^/g, facilitate physical immobilization of metal ions via capillary condensation and multilayer adsorption. Moreover, hydrothermal carbonization at temperatures exceeding 220 °C enhances pore development, resulting in increased pore volumes and, consequently, improved physisorption capacity (Wen et al., 2024). Mesopores in the 3–10 nm range, which are commonly developed in hydrochar produced at HTC temperatures ≥220 °C, promote rapid intraparticle diffusion and effective capillary condensation. These features substantially enhance adsorption performance, often increasing metal uptake capacities by two to fourfold through multilayer physisorption mechanisms. In contrast, pore sizes exceeding ∼4 nm diminishes electrical double-layer overlap, leading to an increase in adsorption enthalpy (ΔH) and a shift toward more endothermic adsorption behavior (Niu et al., 2024). Micropores impose size constraints on hydrated metal ions, for example, Pb^2+^ has an approximate diameter of ∼0.4 nm in its bare form and ∼0.8 nm when hydrated, thereby restricting pore accessibility and preferentially favoring smaller ions such as Cu^2+^ over larger species like Cd^2+^. Although the overlap of surface potentials within micropores enhances ion retention, it also hinders mass transport, resulting in slower adsorption kinetics characterized by reduced intraparticle diffusion coefficient (Suresh Kumar et al., 2019).

Macropores primarily enhance external mass transfer by facilitating rapid solute transport; however, they contribute less than 20% to overall physisorption capacity. Their principal function is to act as transport pathways, enabling efficient access of adsorbates to the micro and mesoporous regions where the majority of adsorption occurs (Strangfeld et al., 2021). Within micropores whose dimensions are comparable to hydrated metal-ion diameters (∼0.4–0.8 nm), van der Waals interactions are significantly amplified due to strong spatial confinement. This confinement shortens equilibrium separation distances by approximately 0.3–0.5 Å and increases dispersion interaction energies by three to fivefold as a result of overlapping surface potentials. Consequently, Hamaker constants increase (A ∝ 1/r), reflecting cooperative interactions arising from the simultaneous influence of electron clouds from opposing pore walls (Eskandari-Ghadi and Zhang, 2022). The presence of oxygen-containing functional groups (–COOH, –OH) decreases the point of zero charge (pH_p_zc) to approximately 4–6, resulting in negatively charged surfaces with zeta potentials of about -15 to -30 mV at pH 5–7. These surface characteristics significantly enhance electrostatic attraction toward cationic metal ions such as Pb^2+^ and Cd^2+^, leading to adsorption capacities that are two- to fourfold higher than those of low-oxygen hydrochar with pH_p_zc > 8. The deprotonation of these functional groups exposes –COO^–^ sites, which play a critical role in ion pairing and cation binding (Zhou et al., 2022). Pb^2+^ removal is dominated by physisorption mechanisms, primarily electrostatic attraction and van der Waals interactions, owing to its relatively small hydrated radius (∼0.4 nm) and strong charge-driven affinity. Consequently, phosphorus-rich hydrochar can achieve exceptionally high adsorption capacities of ∼400–466 mg/g. In contrast, Zn^2+^ removal (>90% at influent concentrations of 10–50 mg L^–1^) occurs mainly through multilayer physisorption and surface ion-exchange processes. Zn^2+^ exhibits a lower adsorption affinity than Pb^2+^ and Cd^2+^ due to its greater hydrolysis tendency and demonstrates optimal performance on hydrochar with high specific surface areas (>200 m^2^/g) (Wallace et al., 2022). Hydrochar synthesized from pulp and paper sludge adsorbed Pb (II) primarily through the synergistic action of oxygen-containing functional groups, ion exchange, electrostatic attraction, and surface complexation. NaOH modification significantly increased the abundance of hydroxyl (–OH) and carboxyl (–COOH) groups as well as the BET surface area, providing more active binding sites for Pb^2+^ ions and enhancing the maximum adsorption capacity from 24.72 to 37.32 mg g^–1^. The adsorption followed the Langmuir isotherm and pseudo-second-order kinetic model, indicating monolayer adsorption dominated by chemisorption through coordination between Pb^2+^ and oxygen donor atoms on the hydrochar surface. In the multi-metal system, Pb^2+^ exhibited the highest adsorption affinity (Pb^2+^ > Cu^2+^ > Cd^2+^ ≈ Zn^2+^) (Nhambe et al., 2025).

### 6.2 Ion-exchange

Hydrochar removes heavy metals via an ion-exchange mechanism, wherein biomass-derived cations (K^+^, Ca^2+^, Na^+^, Mg^2+^) associated with ash minerals are released and replaced by Pb^2+^ and Cd^2+^ from solution. This process is most effective in the pH range of 4–7, where partial mineral dissolution promotes stoichiometric cation exchange, yielding adsorption capacities of approximately 200–350 mg/g. Hydrochar with high mineral content (>10 wt% ash) exhibit two to threefold greater ion-exchange capacity, further enhanced under mildly acidic conditions due to surface protonation. The exchange selectivity typically follows the order Pb^2+^ > Cd^2+^ > Zn^2+^, governed by differences in charge density and hydrated ionic radius compatibility (Wang et al., 2023). Surface functional groups, such as the carboxyl (–COOH) and phenolic hydroxyl (–OH) moieties, promote ion exchange by providing active sites that release protons or associated metal cations (e.g., Ca^2+^, K^+^) upon binding heavy metal ions (Yang et al., 2019). High-ash hydrochar (>10 wt%) provide abundant exchangeable cations such as Ca^2+^, K^+^, and Mg^2+^, primarily originating from carbonate and phosphate minerals, which directly increase the availability of ion-exchange sites for heavy metal uptake. This enhancement has been consistently demonstrated in studies involving mineral-rich biomass-derived hydrochar produced via hydrothermal carbonization (Khosravi et al., 2021). Oxygen-containing functional groups on hydrochar surfaces, particularly carboxyl and phenolic groups, further help ion exchange by deprotonating at pH between 4 and 5, releasing H^+^ and producing negatively charged sites strongly binding heavy metal cations (Rani et al., 2025). Exchangeable Ca^2+^, Mg^2+^, K^+^, and Na^+^ associated with carbonates, phosphates, and oxides in hydrochar ash actively drive ion-exchange reactions with heavy metals such as Pb^2+^, Cd^2+^, and Hg^2+^ (Hua et al., 2020). The grafting of xanthate (-CSS^–^) groups introduced abundant sulfur-containing ligands with a strong affinity for soft metal ions, particularly Pb^2+^, thereby significantly increasing the adsorption capacity. The excellent Langmuir fit suggests that the grafted xanthate (-CSS^–^), hydroxyl (-OH), amino (-NH_2_), and Fe-O groups acted as specific adsorption sites where each metal ion occupied a finite number of binding locations through chemisorption. In the multi-metal system, the Langmuir maximum adsorption capacities were 105.74 mg g^–1^ for Pb^2+^, 39.38 mg/g for Cu^2+^, 38.11 mg/g for Cd^2+^, and 26.53 mg/g for Zn^2+^, demonstrating the highest affinity of the modified hydrochar toward Pb^2+^ (Lin et al., 2024). A phosphate-modified hydrochar from poplar sawdust was evaluated for its performance in the simultaneous removal of Pb (II) and ciprofloxacin from aqueous solution. The phosphate modification substantially increased the density of oxygen- and phosphorus-containing functional groups, resulting in a maximum Pb (II) adsorption capacity of 119.61 mg/g. In the binary contaminant system, the coexistence of Pb (II) and ciprofloxacin produced a synergistic adsorption effect, whereby Pb (II) adsorption was enhanced, while ciprofloxacin adsorption increased at low Pb (II) concentrations but declined at higher metal concentrations because of competition for adsorption sites. Spectroscopic characterization and density functional theory calculations revealed that surface complexation with phosphate groups, precipitation, and π–π interactions dominated Pb (II) adsorption, whereas hydrogen bonding, pore filling, and electrostatic interactions primarily governed ciprofloxacin uptake (Qin et al., 2023).

### 6.3 Redox transformation

Redox processes are very important in the treatment of heavy metals, since they change metal oxidation states and control speciation, solubility, mobility and toxicity. Hydrochar, particularly, can induce such oxidation-reduction reactions using functional groups that change the environmental behavior of heavy metals. Hydrochar can also serve as electron donors themselves or as catalytic surfaces that promote redox cycling and thus reduce metal bioavailability and environmental impact (Wang et al., 2020). These redox and reduction pathways reduce the rate of remediation by turning contaminants into less toxic, less mobile species which reduce environmental and human health risks. Hydrochar has many electron-donating functions, and stable organic matrices that function efficiently during such transformations and provide favorable conditions for environmentally positive redox reactions (Cheng et al., 2020). In particular, hydrochar facilitates the reduction of high-valence heavy metals such as Cr (VI) via electron transfer from oxygen-containing functional groups such as phenolic hydroxyl (–OH) and carbonyl (C=O), which provide electron donors upon initial electrostatic attraction of anionic species such as HCrO_4_^–^ onto protonated surface sites. The redox pathway turns highly toxic and mobile Cr (VI) into less soluble and less toxic Cr (III) (Xia et al., 2019). Amino-functionalized hydrochar (NMHC) prepared from garden-waste biomass effectively removes Cr (VI) by coupling electrostatic adsorption and reductive transformation. Initially, HCrO_4_^–^ anions are attracted to protonated nitrogen functionalities on the hydrochar surface, followed by electron transfer from phenolic hydroxyl and carbonyl groups, which reduces toxic Cr (VI) to the less harmful Cr (III). This process yields a high adsorption capacity of approximately 245 mg/g (Zhang et al., 2025). The removal of Cr (VI) by lignocellulosic biomass-derived hydrochar occurs via a coupled adsorption-reduction-photocatalytic mechanism. Initially, Cr (VI) oxyanions are electrostatically attracted to the protonated hydrochar surface. Subsequently, biomass-derived redox-active functional groups including phenolic, hydroxyl, and quinone-like moieties donate electrons, reducing Cr (VI) to Cr (III). Upon light irradiation, photoexcited electrons generated within the conjugated carbon framework further accelerate the reduction process. The resulting Cr (III) is then immobilized through inner-sphere surface complexation and/or precipitation as Cr (OH)3, ensuring efficient and stable chromium detoxification (Luo et al., 2023).

## 7 Economic feasibility and life cycle assessment

hydrochar is often regarded as a cost-effective material due to its production from low-value biomass, its economic feasibility depends on multiple factors beyond feedstock availability. Recent techno-economic analyses indicate that reactor capital costs, energy consumption, operating conditions, hydrochar yield, and product quality influence HTC economics. Economic viability can be enhanced through process integration, such as coupling HTC with anaerobic digestion for process water management and adopting a biorefinery concept that valorizes hydrochar and other co-products. Furthermore, although HTC has demonstrated economic potential and has reached industrial implementation in selected applications, additional large-scale techno-economic and life-cycle assessments are required to support its widespread commercialization (Cavali et al., 2025). While hydrochar is frequently described as a low-cost adsorbent, this claim should be qualified against the capital and operating costs of the hydrothermal carbonization (HTC) process itself. Process-design and techno-economic studies indicate that the production cost of pelletized hydrochar typically falls in the range of €150–200 per ton, with a break-even price near €200 per ton, depending on feedstock moisture content, reactor temperature (180–250 °C), and residence time (1–8 h) (Lucian and Fiori, 2017). At optimized conditions, thermal energy and electrical power consumption were estimated at roughly 1170 kWh and 160 kWh per ton of hydrochar, respectively, corresponding to an overall plant efficiency of about 78% (Lucian and Fiori, 2017). Minimum production cost of HTC-derived carbon material was 0.0063 USD/MJ, which was 23.2% lower than that of slow pyrolysis. Although HTC showed relatively higher impacts in some individual environmental categories, its overall life-cycle environmental burden was considerably lower. The economic performance of HTC was found to depend primarily on reactor investment, energy consumption, feedstock properties, and operating conditions. Moreover, uncertainty analysis demonstrated that HTC has a 71.8% probability of being the environmentally preferred technology, supporting its potential for future commercial-scale implementation (Li and Jin, 2025). Life cycle assessment (LCA) provides a comprehensive framework for evaluating the environmental sustainability of hydrochar production by considering the entire process from biomass collection to product utilization and end-of-life management. The environmental impacts of hydrochar are influenced by feedstock characteristics, HTC operating conditions, energy requirements, and the treatment of process water and gaseous by-products. Product valorization, particularly through environmental remediation, soil amendment, and carbon sequestration, can significantly improve the environmental performance of HTC systems (Ogunleye et al., 2024). Unlike commercial activated carbon, whose regeneration (thermal or chemical) is well-characterized and costed, regeneration protocols for spent hydrochar remain comparatively underexplored, and repeated adsorption-desorption cycling can degrade surface functional groups and reduce adsorption capacity, adding uncertainty to long-term operating cost estimates. Where post-synthesis activation (chemical or physical) is used to enhance hydrochar’s adsorption performance, this step adds further capital and reagent costs that should be included in any cost-per-unit-pollutant-removed comparison (techno-economic considerations of hydrochar as an adsorbent depend on feedstock type, water-to-biomass ratio, temperature, residence time, and post-treatment activation needs) (Selvaraj et al., 2025).

## 8 Bacterial remediation of heavy metals

**FIGURE 2:**
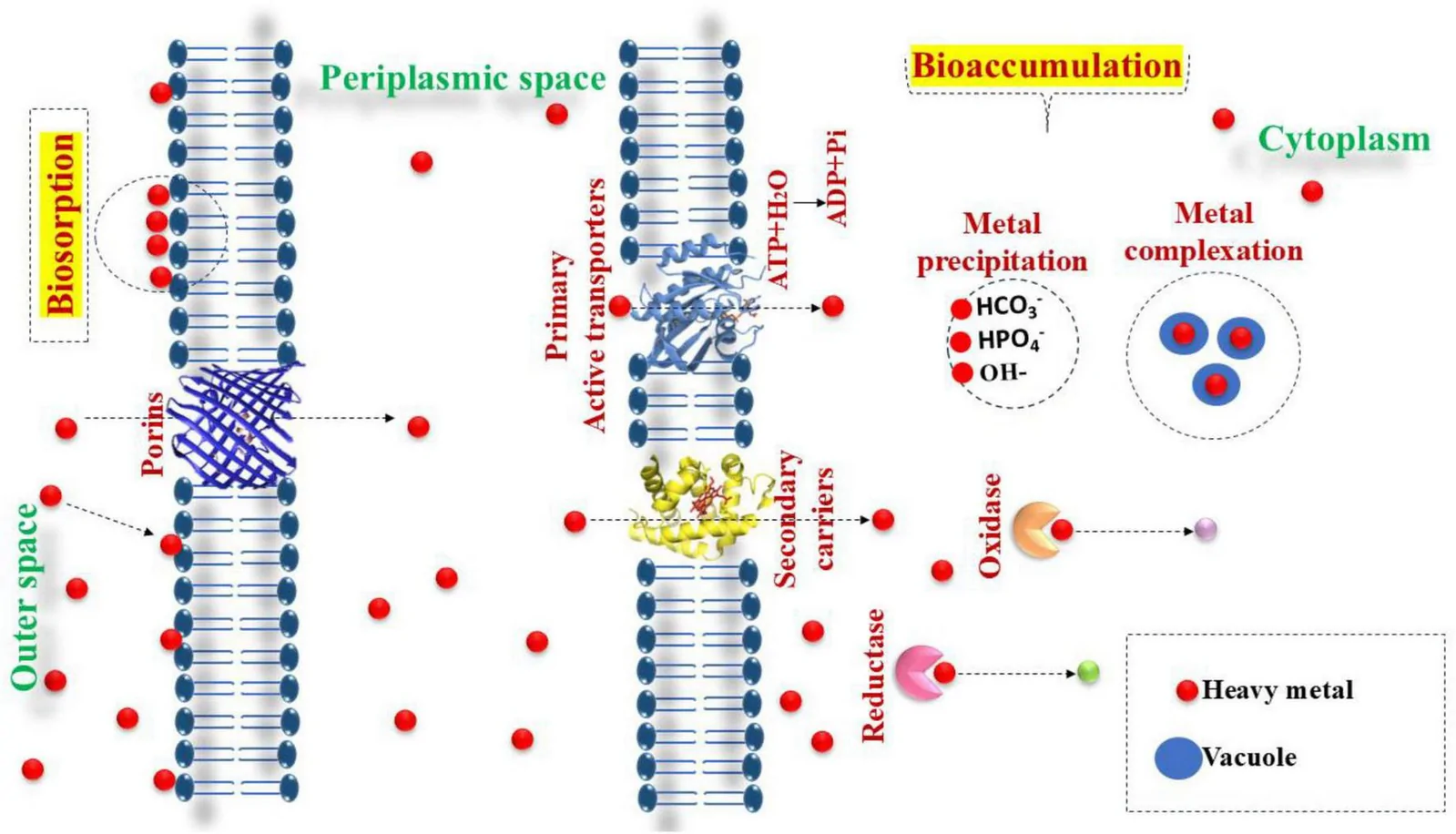
Bacterial heavy metal remediation mechanism.

Bacteria have adopted various mechanisms in the remediation of heavy metals, and they include biosorption, intracellular accumulation, and bio-transformation (Figure 2). The cell wall structure of bacteria is highly ordered, mesh-like, and critical in maintaining cell morphology and structural stability. The cell wall structure mainly comprises peptidoglycan, which offers mechanical strength and protection to the cell (Dörr et al., 2019). The basic structural building blocks of peptidoglycan are its glycan chains, which are cross-linked with peptide bridges. The glycan chains are made up of alternating N-acetylglucosamine (GlcNAc) and N-acetylmuramic acid (MurNAc) units, linked through a β-1,4-glycosidic bond. Each MurNAc unit is attached to a D-lactoyl moiety, which is then replaced by a peptide stem. The peptide stem is generally made up of A_2_pm, 2,6-diaminopimelic acid. Cross-links are generally made between the carboxyl groups of the D-Ala at the 4th position and the amino groups of the diamino acid at the 3rd position, directly or through a peptide bridge. The chemical composition that defines this heteropolymer includes the presence of the unusual sugar MurNAc, γ-linked D-glutamate, and peptide bonds involving L-D (and rarely D-D) amino acids, as well as non-proteinogenic amino acids (Vollmer et al., 2008). Gram-positive organisms are characterized by the presence of a cell envelope with multiple layers of peptidoglycan, which are thicker than those found in the cell envelope of *Escherichia coli*. Present within these layers are long chains of anionic polymers called teichoic acids, which are mainly composed of repeating units of glycerol phosphate, glucosyl phosphate, and ribitol phosphate. One of the teichoic acids is called wall teichoic acids and is covalently linked to the peptidoglycan matrix and contributes to the total negative charge of the cell surface. Gram-positive bacteria like *Staphylococcus aureus* and *Bacillus subtilis* are characterized by the presence of teichoic acids. On the other hand, the outer membrane (OM) is composed of phospholipids located on the inner leaflet of the OM, while the outer leaflet is composed of glycolipids like lipopolysaccharides (LPS) (Silhavy et al., 2010). Collectively, these structural features make bacterial cells highly effective for heavy metal remediation.

### 8.1 Biosorption

Intrinsic capacity of microorganism is explored in biosorption to sequester and immobilize heavy metals from pollutant environmental matrices, thereby offering a sustainable, efficient, and cost-effective alternative to conventional remediation technologies (Koller and Saleh, 2018). In parallel, Beveridge and colleagues established the structural basis of bacterial metal binding, showing that the bacterial cell wall itself particularly peptidoglycan and associated anionic groups could immobilize soluble metals independent of cellular metabolism, laying the mechanistic groundwork for treating bacterial cell walls as natural ion-exchange matrices (Beveridge and Murray, 1976). One of the most important economic benefits of biosorption technology is the fact that this technology relies on the abundance of naturally occurring low-cost biomass materials. These materials tend to be more efficient and sustainable compared to conventional remediation techniques. Moreover, this environmentally friendly technology has the capacity to remove a wide range of heavy metals from the environment without the production of hazardous by-products (Aslam et al., 2025). Adsorption is characterized by non-specific physicochemical interactions between organic or inorganic substances and microbial cell components. The adsorption of certain heavy metals is mainly governed by the interaction between active functional groups present on the microbial surface and the heavy metal ions. Electrostatic forces control the interaction with the charged species, while van der Waals forces control the interaction with the uncharged species. Physical adsorption is mainly governed by van der Waals forces, while chemical or activated adsorption is governed by the specific interaction between the adsorbent and the adsorbate (Jhariya et al., 2025). The enhanced sorption capacity of Gram-positive bacteria has been primarily ascribed to the presence of the thick peptidoglycan-rich cell wall (Pham et al., 2022). The cell wall of Gram-negative bacteria has been observed to be relatively thinner compared to the cell wall of Gram-positive bacteria. The cell wall of Gram-negative bacteria consists of a monolayer of peptidoglycan and an outer membrane consisting of lipopolysaccharides (LPS), phospholipids, and surface proteins. The phosphate functional groups present in the LPS and phospholipids have been observed to be the main binding sites for the interaction of metal ions (Yadav et al., 2018).

Ion exchange is a key biosorption process in which incoming heavy metal ions, such as Pb^2+^, replace lighter cations such as H^+^, Na^+^, and Ca^2+^, which are present as ions with functional groups such as carboxyl and hydroxyl on the bacterial cell surface and EPS (Wei et al., 2016). The heavy-metal-resistant *Bacillus* isolate (strain WW6) recorded the highest Pb^2+^ biosorption capacity in the unary system at 0.36 mmol g^–1^. However, its capacity progressively reduced in the binary systems with Ni^2+^ at 0.25 mmol g^–1^ and Cd^2+^ at 0.22 mmol g^–1^, and finally in the ternary Pb^2+^-Cd^2+^-Ni^2+^ system at 0.21 mmol g^–1^ due to the presence of competing heavy metal ions. In addition, the optimal pH for lead removal varied in different systems, from 7.5 in the unary lead system, 6 in the Pb^2+^-Ni^2+^ system, 5.5 in the Pb^2+^-Cd^2+^ system, and 6.5 in the ternary Pb^2+^-Cd^2+^-Ni^2+^ system. Heavy metal ions at high pH values above 9 form precipitates in aqueous solutions, thus reducing their concentration in the solution and hence reducing the efficiency of removal from the aqueous phase. On the contrary, at acidic pH values, there is competition for active sites on the surface of the bacteria between heavy metal ions like Pb^2+^, Cd^2+^, and Ni^2+^, and H^+^ ions (Ahmady-Asbchin et al., 2024). In the study on *Bacillus altitudinis* CdRPSD103, it was found that this halophilic bacterium was able to remove heavy metals through cell surface biosorption, which follows pseudo-second-order kinetics and Langmuir isotherm. The detoxification of cadmium occurs through biotransformation, where cadmium is converted to cadmium sulfide, which is less toxic and insoluble. The formation of yellow-green precipitates is an indication of cadmium sulfide synthesis, which occurs after six hours of incubation with cadmium ions. This is facilitated by sulfhydryl groups, which are present in cysteine. The sulfhydryl groups are responsible for binding cadmium ions to form cadmium sulfide nanoparticles. The role of thiol groups is very important in synthesizing cadmium sulfide nanoparticles, as it is responsible for creating stable complexes with heavy metals. The antioxidant property of thiol groups is responsible for scavenging free radicals. However, heavy metals such as cadmium, silver, and mercury can inhibit enzyme activity by binding to thiol groups. Negatively charged groups such as S^2–^ and PO4^3–^ present on the surface of metal-resistant bacteria can readily interact with cadmium ions, thereby reducing Phyto availability (Usmonkulova et al., 2025).

The *Bacillus cereus* SEM-15 strain demonstrated a high potential for inorganic phosphorus solubilization, indole-3-acetic acid (IAA) production, and lead uptake. Fourier transform infrared (FTIR) spectroscopy indicated the presence of new peaks at 541.68 and 980.24 cm^–1^ after lead adsorption, attributed to S-S and Pb-S bonds, respectively. These bonds indicate the involvement of disulfide bonds and Pb-S complexes in the adsorption process. Genomic sequence analysis also showed the presence of metal resistance genes, such as the cadmium efflux pump genes *cadA* and *cadC*, the *czcD* gene associated with the Czc efflux system, and the *corA* gene associated with the Cor transport system (Li et al., 2023). Furthermore, *Bacillus cereus* AUMC B52 was able to display high capacity for Zn (II) biosorption, reaching a maximum adsorption capacity of 66.6 mg g^–1^ as calculated by the Langmuir isotherm model. This is largely due to the presence of specific functional groups like amine, hydroxyl, carboxyl, and carbonyl (Joo et al., 2010). The study on S-layer metal interaction was aimed at investigating the interaction of metal ions with the S-layers of *Lactobacillus kefir* CIDCA 8348 and *Lactobacillus kefir* JCM 5818 using TEM and FTIR. The study showed that cadmium induced precipitation in the S-layers and caused significant structural changes upon adsorption. These structural changes involve alterations in the structure of the carboxyl group in aspartic acid (Asp) and glutamic acid (Glu) residues, as well as alterations in the structure of some N–H group peptide backbone. Additionally, the study showed alterations in the secondary structure of the S-layers, where the α-helix structures were altered and β-sheet structures were formed, which might contribute to the energy minimization process (Gerbino et al., 2011).

### 8.2 Bioaccumulation

Bioaccumulation occurs when the rate of pollutant uptake exceeds the rate of its elimination, leading to the intracellular retention and progressive buildup of contaminants within the organism. This process requires metabolically active (living) cells, as pollutant uptake is driven by energy-dependent mechanisms. Bioaccumulation proceeds in two distinct stages: an initial, metabolically independent phase involving the passive binding of metal ions to the cell surface, followed by a second, metabolically active phase in which the bound metal ions are actively transported into the cytoplasm for intracellular sequestration (Nnaji et al., 2023). Metal accumulation occurs through the action of specific metal transport systems (influx and efflux mechanisms), along with intracellular precipitation and complexation processes within the bacterial cell (Abo-Alkasem et al., 2023). During complexation, microorganisms especially bacteria and cyanobacteria employ intracellular metal-binding proteins such as metallothioneins to sequester metal ions, thereby preventing free metals from interacting with and damaging cellular components (Nnaji et al., 2024).

A variety of bacterial species, such as *Bacillus*, *Pseudomonas*, and *Enterobacter*, as well as other microorganisms found in soil, have been found to accumulate cadmium through energy-dependent mechanisms. Once inside the cells, cadmium is stored through mechanisms that include metallothioneins, metal-binding proteins, and storage in cellular structures. Additionally, cadmium resistance genes such as *cadA* contain P-type ATPases that are ATP-dependent metal transporters, thereby facilitating cadmium homeostasis within the cells (Galan-Freyle et al., 2025). Certain bacterial strains, such as *Cupriavidus taiwanensis* KKU2500-3 and *Pseudomonas aeruginosa* KKU2500-8, have been found to have the capability to precipitate cadmium in the form of cadmium sulfide (CdS) under both aerobic and strictly anaerobic environments. The biomineralization of cadmium is achieved through the production of sulfide, which is a result of the action of cysteine desulfhydrase on D-cysteine, producing hydrogen sulfide (H_2_S). The H_2_S then combines with soluble cadmium ions to form precipitates of cadmium sulfide. The biomineralization of cadmium is normally associated with bacterial cell envelopes, mainly occurring in the periplasm (Jebril et al., 2022).

It has been reported that in the case of *Escherichia coli*, the transport of Ni^2+^ ions are mediated by an ATP-binding cassette (ABC)-type nickel permease system named NikABCDE. The transport of Ni^2+^ is regulated by the nickel-responsive transcriptional regulator NikR. The NikR binds to the promoter region of the nikABCDE operon in the presence of nickel ions. This regulatory mechanism prevents excessive intracellular accumulation of Ni^2+^, thereby enabling *E. coli* to maintain metal homeostasis and tolerate nickel at controlled levels (Deng et al., 2013). The transport of heavy metals into bacterial cells occurs through membrane-associated systems, such as channels, secondary carrier proteins, and primary active transport. Channel proteins are usually α-helix proteins that facilitate passive diffusion of metal ions through the membrane according to their concentration gradient. Channel proteins are a member of the major intrinsic protein (MIP) superfamily. Channel proteins have been known to transport metals such as arsenic and mercury in bacteria such as *Escherichia coli*, *Corynebacterium*, *Streptomyces coelicolor*, *Serratia*, and *Pseudomonas*. In Gram-negative bacteria, β-barrel proteins and porins are also known to play a role in the transport of heavy metals through the outer membrane. Secondary carrier proteins such as uniporters, symporters, and antiporters play a significant role in metal uptake and accumulation. Transporter-opsin-G protein-coupled receptor (TOG) superfamily and major facilitator superfamily (MFS) are known to play a role in metal uptake through secondary carrier proteins. The uptake of metals such as nickel, cobalt, and arsenic has been documented in bacteria such as *Helicobacter pylori*, *Rhodopseudomonas palustris*, *Novosphingobium aromaticivorans*, *Staphylococcus aureus*, and others (Sreedevi et al., 2022). In bacteria, metal efflux transporters are mainly mediated by three major families of exporters: cation diffusion facilitators (CDF), resistance-nodulation-cell division (RND) efflux pumps, and P-type ATPases. Of these, CDF proteins are considered to belong to one of the most common protein families in all living organisms. P-type ATPases are known to play a significant role in metal ion transport across the bacterial membrane through ATP hydrolysis-dependent active transport mechanisms.

A well-characterized metal efflux system in bacteria is *ZntA* from *Escherichia coli*, a P-type ATPase that plays a key role in zinc efflux. Its expression is mediated through a MerR transcriptional regulator, ZntR. When there are higher levels of intracellular zinc ions than the sub-toxic threshold, the apo-ZntR dimer binds to the promoter region of the *zntA* gene, thereby modulating its transcription (Hussain et al., 2022). Both Gram-positive and Gram-negative bacteria, when exposed to iron-limited growth conditions, secrete siderophores, which chelate Fe^3+^ and help it enter the bacteria. In Gram-positive bacteria, ferric siderophore complexes are transported across the membrane by protein-bound systems. On the other hand, Gram-negative bacteria employ an outer membrane receptor, a periplasmic binding protein, and an ATP-binding membrane transporter. After entering the cytosol, Fe^3+^ is reduced to Fe^2+^ and released, whereas the siderophore is degraded or recycled and removed by efflux systems. Hydroxamate siderophores are one of the most common siderophore types found in nature. These are synthesized by a variety of bacteria and fungi. Hydroxamate siderophores include a functional group with a C(=O)–N(OH)-R group, where R is an amino acid or a derivative of an amino acid. Two oxygen atoms coordinate with Fe^3+^, forming a stable complex with an octahedral configuration. Similarly, catecholate siderophores coordinate with Fe^3+^ with a high degree of stability through two oxygen atoms per catecholate group. These catecholate siderophore complexes are stable with a high degree of affinity. Carboxylate siderophores, synthesized by bacteria such as *Rhizobium* and *Staphylococcus*, and fungi belonging to the *Mucorales* group, coordinate with Fe^3+^ through a carboxyl and a hydroxyl functional group (Řezanka et al., 2018). The enzymatically synthesized HPO_4_^2+^ by *Citrobacter* plays a crucial role in metal precipitation in the form of insoluble metal phosphates. Further, the synthesis of H_2_S by SRB (Sulfate reducing bacteria) plays a crucial role in metal precipitation in the form of metal sulfides, which acts as an alternative strategy for metal-contaminated effluent treatment (Hansda et al., 2016). Copper homeostasis in bacteria involves the cop operon, which encodes proteins involved in copper transport and detoxification. CopB acts as an outer membrane protein involved in copper uptake in the periplasm. It plays a crucial role in regulating excess Cu^+^/Cu^2+^ ions. In the periplasm, copper ions are bound by CopA, which prevents copper from entering the cytoplasm. CopC and CopD play a crucial role in transferring copper ions from the periplasm to the cytoplasm, where it acts as a cofactor for metalloenzymes (Irawati et al., 2021).

### 8.3 Biotransformation

Mercury biotransformation was the first and most thoroughly studied system historically, beginning with the discovery of mercurial resistance in clinical R-factor-bearing isolates and the subsequent purification of a mercuric-ion-reducing enzyme from *Escherichia coli* carrying resistance plasmids the mechanism of mercuric chloride resistance in microorganisms was elucidated through purification of a mercuric ion reducing enzyme from *E. coli* bearing an R factor, alongside NADPH-dependent reduction of mercuric chloride and vaporization of mercury by a multiple-drug-resistant strain of *E. coli*. This enzymatic detoxification converts toxic mercurials into volatile species such as methane, ethane, or metallic mercury that are rapidly lost from the environment, while cadmium and arsenate resistance instead operate through reduced net cellular accumulation the mechanism of mercury and organomercurial resistance is enzymatic detoxification of the mercurial into volatile species which are rapidly lost from the environment, while cadmium and arsenate resistances are due to reduced net accumulation of these toxic materials. Parallel to bacterial mercury reduction, early aquatic microbiology established that bacteria could also *methylate* mercury, forming the highly bioaccumulative and neurotoxic methylmercury species central to environmental mercury cycling formation of methylmercury (Silver, 1984).

One of the most common mechanisms for the bacteriological depletion of heavy metals is the activation of various redox enzymes for the generation of less toxic or less mobile forms of highly toxic metal species. The various bacteriological oxidoreductases play a significant role in the reduction of the concentration of metals like Cr (VI), Hg (II), and As (V) by reducing the activities of the corresponding chromate reductases, mercuric reductase (merA), and arsenate reductase (arsC). In addition, microbial methylation/demethylation is also a significant mechanism for the changes in the redox potential. All these processes play a significant role in the sustainable bioremediation of metals by inhibiting the bioaccumulation of metals (Ding et al., 2024). Certain bacteria, including *Micrococcus* spp. and *Acinetobacter* spp., have been reported to oxidize arsenic, thereby converting it into less toxic and less mobile forms (Nagvenkar and Ramaiah, 2010). One of the important bio-reduction mechanisms is the process of extracellular electron transfer (EET), where electron carriers such as riboflavin or flavin mononucleotide act as electron donors from the bacterial respiratory chain to metals such as U(VI) or Cr (VI), thereby promoting their reduction outside the cell. This is particularly significant under anaerobic conditions. For example, *Shewanella* species utilize c-type cytochromes located in the outer membrane of the cell wall (e.g., MtrC) for the electron transfer process, which links the oxidation of organic matter with the reduction of metals, thereby precipitating these metals as oxides or sulfides (Jayaram et al., 2022). The microbial chromium biotransformation is strictly regulated by the redox reactions related to the iron cycle, as reported in *Shewanella oneidensis MR-1*. *S. oneidensis MR-1* reduces the highly toxic and mobile form of chromium, hexavalent chromium [Cr (VI)], to the less toxic and less mobile form, trivalent chromium [Cr (III)], using extracellular and membrane-associated electron transfer mechanisms. Moreover, the organism promotes the reduction of ferrihydrite-bound Cr (VI) (Hu et al., 2024). *Rhodococcus rhodochrous* As33 exhibit a potent chromate reductase activity, capable of reducing the highly toxic hexavalent chromium [Cr (VI)] to the less toxic trivalent chromium [Cr (III)] through the action of intracellular and membrane-associated reductase enzymes. Thus, the major mechanism for the removal of chromium is the action of the enzymes, rather than adsorption (Gf et al., 2025). Methylation is one of the most important biochemical processes in bacteria that is closely associated with the transformation and detoxification of metals and metalloids. Intracellular methylation occurs through different pathways using S-adenosylmethionine (SAM), methylcobalamin (an active vitamin B12 compound), and N-methyltetrahydrofolate. Sulfate-reducing and iron-reducing bacteria commonly use methylation pathways mediated by methylcobalamin for mercury methylation to produce highly toxic and volatile methylmercury compounds (CH_3_Hg^+^). In contrast, methylation of arsenic compounds is mediated by SAM-dependent pathways to produce volatile arsine gas (AsH3) (Chakraborty et al., 2026). The reduction of hexavalent chromium to the less toxic trivalent state is mediated by two groups of enzymes: specific chromium reductases like Chr, NemA, and YieF, and nitroreductase-associated enzymes like YcnD, NfsA, and NfsB. The reduction is NADPH-dependent, with NADPH acting as the primary electron donor for this reaction. In aerobic bacteria, Cr (VI) reduction occurs in the presence of oxygen, with Cr(V) and Cr (IV) as reactive intermediates in the formation of Cr (III). In this reaction, ROS like H_2_O_2_, O_2_^–^, and –OH are also produced, leading to oxidative stress in the bacterium. Antioxidant enzymes like catalase (CAT), superoxide dismutase (SOD), and glutathione S-transferase (GST) are produced in response to this oxidative stress. In contrast, in anaerobic bacteria, Cr (VI) reduction occurs in the absence of oxygen, with minimal production of ROS. In both cases, Cr (III), which is produced in this reaction, is effluxes out from the bacterium (Ramli et al., 2023). Flavin reductase (Fre) in *Escherichia coli* facilitates the reduction of Cr (VI) to a soluble Cr (III)-NAD^+^ complex via highly reduced, free flavin intermediates, highlighting its role in enzymatic chromium detoxification (Ahemad, 2014). The acetyl-CoA pathway is an important mechanism for mercury methylation in microorganisms. It consists of a folate branch that provides a methyl group and a carbonyl branch that provides a carbonyl group, both of which result from the reduction of CO_2_. The two branches are then combined by acetyl-CoA synthase to form an acetyl-CoA molecule. The folate cycle is important for the reduction of 5,10-methylenetetrahydrofolate (CH_2_-THF), which is a key step in one-carbon metabolism. Stable isotopic studies have demonstrated that [3-^14^C] serine is converted to CH_2_-THF with 95% incorporation into methylmercury (MeHg), confirming CH3-THF as the primary methyl donor in mercury methylation (Tao et al., 2026).

Remediation genes for mercury (Hg) are often investigated using PCR techniques. The *merA* gene and *merR* gene are the most significant ones. The *merA* gene is responsible for the production of mercuric reductase, an enzyme that reduces mercury ions to mercury vapor using NADPH as an electron donor. The *merR* gene is responsible for the production of a protein that represses the mer operon in the absence of mercury but activates it in its presence (de Mattos D’Avila et al., 2024). Lead can also be precipitated in aerobic as well as anaerobic conditions through redox-mediated reactions. In this case, Pb^2+^ is reduced to Pb^0^ through electron transfer reactions. In anaerobic conditions, different electron donors like nitrate and Sulfate can also mediate this reaction, leading to the formation of ammonia/nitrogen and sulfide, which in turn can combine with Pb^2+^ to form insoluble PbS, which is normally dark gray or black in color. In aerobic conditions, Pb^0^ can also be oxidized to PbO, leading to brown or yellow-colored precipitates. In this case, bacterial action, particularly that of SRB (Sulfate reducing bacteria) is important in converting soluble Pb^2+^ to insoluble forms like Pb^0^, PbO, or PbS, thus limiting its bioavailability or uptake by bacterial cells (Sevak et al., 2021).

### 8.4 Bacterial metal influx and efflux systems

Metal ions such as Fe, Zn, Cu, Mn, Ni, and Co serve as essential cofactors for a large fraction of bacterial enzymes, yet the same ions become cytotoxic when present in excess, generating oxidative stress, mismetalation of proteins, and membrane damage. To reconcile this dual requirement, bacteria have evolved a tightly coordinated network of influx (uptake) and efflux (extrusion) systems, regulated by metal-sensing transcription factors that switch import machinery on during scarcity and efflux/storage machinery on during excess metal limitation activates pathways involved in the import and mobilization of metals, whereas excess metals induce efflux and storage. This regulatory logic is built around protein- and RNA-based metal sensors that detect intracellular metal status and reinforce the response at both transcriptional and post-transcriptional levels recent insights highlight protein-based and RNA-based sensors that interact directly with metals or metal-containing cofactors, with the transcriptional response reinforced by post-transcriptional regulatory systems (Chandrangsu et al., 2017). Many bacteria have an arsenic resistance (ars) operon made up of a grouping of 3 genes: *arsR*, *arsB*, and *arsC*. Each of these genes codes for an individual protein (i.e., ArsR, ArsB, or ArsC), and when all 3 proteins are combined, they represent the minimum genetic requirements for conferring resistance to arsenic. The *arsR* gene codes for a trans-acting transcriptional regulator that works as a repressor to regulate all 3 genes in the operon, including the arsR gene itself. The ArsR protein binds to the DNA via an HTH (helix-turn-helix) domain and has specific amino acid residues that interact with metal oxyanions (i.e., arsenite, arsenate, and antimonite). The presence of additional (more than one) ArsR proteins in some bacteria indicates the possibility of more than one arsenic resistance (ars) operon per bacterium. The *arsB* gene produces the ArsB protein, a membrane associated arsenite efflux pump that transports toxic arsenite from the inside to the outside of the bacterium. In simpler arsRBC operons, the primary energy source driving this efflux is the proton motive force across the membrane. The *arsC* gene produces a glutaredoxin-dependent arsenate reductase that reduces arsenate to arsenite; this allows for the efficient exit of both forms of arsenic from the bacterium using the ArsB transporter (Roy et al., 2024). CadA is a membrane-associated P-type ATPase mainly found in Gram-positive bacteria, e.g., *Staphylococcus aureus*, and plays a critical role in cadmium resistance by actively transporting Cd^2+^ ions out of the bacteria. CadA is a primary active transporter because it utilizes ATP hydrolysis and a transient phosphorylated intermediate, a characteristic feature of P-type ATPases. CadA is composed of several transmembrane alpha-helices for transporting ions, an N-terminal cysteine-rich metal-binding domain, an ATP-binding (kinase) domain containing a conserved aspartate residue for ATP phosphorylation, and a transduction domain for coupling ATP hydrolysis with metal transport. CadA works by binding Cd^2+^ ions within the cytoplasm of the bacteria and then using ATP hydrolysis to actively transport it out of the bacteria, thus maintaining metal homeostasis within the bacteria (Silver and Ji, 1994). On the efflux side, bacteria rely on several structurally and mechanistically distinct transporter families. P-type ATPases use ATP hydrolysis to actively pump mono- and divalent metal cations out of the cytoplasm; RND (resistance-nodulation-cell division) transporters function as tripartite CBA efflux pumps that span both membranes of Gram-negative bacteria; the Cation Diffusion Facilitator (CDF) family acts as chemiosmotic ion/proton exchangers with six transmembrane helices for divalent cation efflux; and additional systems such as ABC transporters, HoxN, CHR, and MIT-family proteins contribute metal-specific transport activity efflux systems and associated transporter proteins integral to heavy-metal homeostasis include P-type ATPases, RND, CDF, ABC transporters, HoxN, CHR, and MIT-family proteins, differing in topology, substrate specificity, and energy-coupling mechanisms. A systematic comparison across 63 sequenced prokaryotes and the model heavy-metal-resistant organism *Ralstonia metallidurans* showed that resistance typically arises from multiple, overlapping efflux systems rather than a single dominant pathway. Some genes broadly conserved for basal defense, others highly specialized and restricted to a few lineages heavy metal resistance results from multiple layers of resistance systems with overlapping substrate specificities but unique functions, some widespread for basic cellular defense and others highly specialized in only a few bacteria (Adhikary et al., 2024).

## 9 Hydrochar-bacteria synergistic system

Hydrochar-bacteria immobilized systems have emerged as an efficient strategy for heavy metal (HM) remediation due to their synergistic interactions. Hydrochar serves as a functional support matrix, providing abundant adsorption sites and a protective microenvironment for microbial activity. The immobilized bacteria enhance metal removal through biotransformation, biosorption, and precipitation mechanisms. Together, this integrated system improves removal efficiency, stability, and resistance to environmental stress, making it a promising approach for sustainable remediation. (Long et al., 2024) carried out an experiment that determined the effect of adding hydrochar produced from dairy manure to the composting process involving swine manure and wheat straw in relation to the stabilization of heavy metals. Results show that the hydrochar provides a better micro-environment and thus leads to a prolonged thermophilic stage as well as changes in the bacterial community composition. These changes facilitate humification leading to increased concentration of humic acid. This results in low levels of bioavailable heavy metals such as copper, zinc, lead, and nickel due to increased molecular complexation and microbial transformation processes. Hydrochar is a new form of carbonaceous material possessing a high specific surface area (4.9 m^2^/g) with many oxygen containing functional groups, e.g., amide group (C=O), aryl ether (C–O), ester group (C–O), and polysaccharide (C–O). The properties of hydrochar lead to HM passivation via adsorption and surface complexation mechanisms among others. In addition, the use of hydrochar leads to HA formation. The diverse functional groups present in HA, particularly carboxyl and phenolic hydroxyl groups, further decrease the mobility and transformation of HM ions via complexation, adsorption, and redox reactions (Long et al., 2024).

The nitrated hydrochar (NHC) immobilizes the Cd through physicochemical and biological processes together. The use of nitric acid leads to the addition of various surface functional groups which increase its ability to adsorb and therefore stabilize Cd using processes such as adsorption, complex formation, ion exchange, and precipitation, leading to the conversion of bio-available Cd, EXC-Cd, to OM-Cd and CA-Cd. It also helps control the pH levels of the soil, limiting the mobility of Cd. On the other hand, it alters the microbial communities in terms of the dominance of Proteobacteria and Firmicutes over Acidobacteria and increases network complexity of microbes. Specific microorganisms that enhance immobilization include *Anaerolineae*, *Desulfovibrionaceae*, *Burkholderiaceae*, and *Rhodobacteraceae* through processes of sulfide precipitation and organic complexation (Wu et al., 2023). The removal of Cd through hydrochar-bacteria systems involved both physicochemical processes and biological processes through microbe-metabolism. The application of hydrochar in combination with CaO-modified hydrochar involved the use of hydrochar produced through hydrothermal carbonization from biomass precursors (agricultural residues or manure) which was then modified with CaO and had high adsorption efficiency as well as higher levels of alkalinity. Hydrochar was able to adsorb Cd through chemical complexation, ion exchange, precipitation reactions, as well as through microbes which were able to transform metals. In addition to this, it was able to change soil pH, nutrients, microbial community composition toward Proteobacteria and Firmicutes, carbon metabolism and nitrogen metabolic pathways thus decreasing Cd. Moreover, the incorporation of hydrochar also allowed for the introduction of labile carbon into soil which was easily used up by soil microbes, improving their metabolisms. In terms of functional prediction using PICRUSt database, the major KEGG pathways involved were Metabolism (78% to 80%), Genetic Information Processing (6.6% to 6.7%) and Environmental Information Processing (4.9% to 5.2%). Carbohydrate metabolism and amino acid metabolism were dominant within Metabolism pathway. Nevertheless, CaO-hydrochar greatly boosted such biological processes, suggesting increased microbial activities and interactions with Cd. Moreover, the pathways involving glyoxylate and dicarboxylate metabolism became more prominent, leading to better Cd transport control and tolerance. Increased amino acid exudation would then enhance the chelation process, enable the formation of Cd complexes and increase plant tolerance to Cd stress (Lang et al., 2024).

Hydrochar was obtained from As(V) contaminated ramie straw at various temperatures using the alkali modified method of sodium hydroxide addition to obtain pure hydrochar. Using alkali-modified HC in As-contaminated sites improved the soil pH and SOM (Soil organic matter) making a suitable microbial environment for possible dominance of phyla and thus mobilizing soil arsenic. Alkaline modification improves the pH of HC itself. Also, HC is capable of binding H + ions to its surface through its negatively charged functional groups thus forming alkali salts which could be released in the soil. Moreover, HC increases SOM by freeing its fixed carbon content and humic substances, hence promoting humification reactions. The increase in soil pH due to HC addition makes the surface of the soil negatively charged (OH-) making it easier for repulsion of arsenic. HC increased significantly the relative abundance of the dominant phyla, such as Proteobacteria, Patescibacteria, and Acidobacteria, and dominant fungal phyla Mucoromycota. Proteobacteria, As(V) respiring bacteria, can increase the anaerobic reduction of As(V) through the *arrA* gene, thereby releasing As (III) into the pores and mobilizing the accumulation of Arsenic (Qiu et al., 2024). Moreover, Wang et al. (2022) conducted another study on the effectiveness of calcium-modified hydrochar (HC-Ca) on increasing the efficiency of the microbial reduction of hexavalent chromium (Cr (VI)). This process was carried out by using HC-Ca as a carrier immobilizing the bacteria of *Priestia megaterium* strain BM.1. This resulted in a synergistic composite material called BM.1-Ca. As per the sources, the removal efficiency reached 97% at the initial Cr (VI) concentration of 60 mg/L, which was 1.96 times increase from the use of bacteria alone. Furthermore, according to adsorption kinetics, the removal of Cr (VI) by BM.1-Ca is based on pseudo-second-order kinetics, indicating the chemisorption-controlled process. In addition, the Cr (VI)-reducing bacterium, BM.1, actively reduces Cr (VI) to Cr (III) via metabolic reduction. At the same time, the EPS secreted by the bacteria enables the adsorption of reduced Cr (III). The bacterial activity can take place within a hydrochar-based carrier (HC-Ca), which ensures a suitable environment and carbon source and prevents the toxicity of Cr (VI). Moreover, the high abundance of surface functional groups (–OH, C=O, C–O, N–H, P–O) helps remove the metals through complexation and ion exchange mechanisms (Wu et al., 2025).

## 10 Challenges

The coupling of hydrochar with bacteria gained sheer recognition among scientific community due to its effective synergistic performance for heavy metal removal. Despite, several operational challenges must be overcome to make large scale application feasible.

1. One major limitation to be addressed is the harmonization between hydrochar and microorganisms, as hydrochar may contain residual organic compounds that can adversely affect bacterial viability and metabolic activity.
2. Prevalence of microporosity in hydrochar matrices hampers adhesion of bacteria and limits mass transfer of nutrients and metal ions.
3. In complex wastewater system, competition among co-existing metal ions for adsorption sites and hindrance from dissolved organic and inorganic salts further diminishes removal efficiency and selectivity.
4. Sustaining redox environments for microbial metal transformation remain challenging, especially in dynamic environmental conditions.
5. Long term functional integrity of hydrochar-bacteria system is not fully established due to saturation of adsorption site, metal desorption under pH and decline in bacterial viability, alongside environmental risk associated with metal-laden hydrochar, potential release of fine carbon particles, and ecological risks associated with microbial dissemination, also require careful evaluation.
6. Use of waste biomass as a precursor to produce hydrochar results in variability in structural composition, thereby inconsistent adsorption properties across batches, which challenges the large-scale production of hydrochar-bacteria composite

## 11 Conclusion and future perspective

Hydrochar has recognized as a promising and effective material for mitigating heavy metal contamination, offering a sustainable solution to address critical environmental pollution challenges. The role of hydrochar in hydrochar-bacteria synergistic system as an augmentation tool as well as bacterial support matrix have discussed in this paper. The core mechanism behind the hydrochar adsorption involves physical adsorption, ion-exchange, complexation and reduction where as bacteria employs biosorption, bioaccumulation and bio-reduction. Optimal hydrochar characteristics such as enhanced surface area and porosity, surface functionalities are achieved by synthesizing at optimized reaction temperature, pressure, biomass-water ratio and reaction time. The interaction between hydrochar and bacteria enhances the efficiency of metal remediation. The constraints associated with bacterial remediation can be mitigated by hydrochar, which enhances microbial survival and activity by offering nutrients and a protective habitat through its elemental composition and porous structure. Moreover, the bacteria-hydrochar integrated system represents a cost-effective and environmentally sustainable strategy for the remediation of hazardous metal ion contamination.

Future studies can emphasize on following aspects.

1. Detailed study on the ecotoxicological effect of hydrochar, durability and synthesize optimization is necessary.
2. Establishment of guidelines on handling and disposal of hydrochar is essential.
3. It is imperative to optimize the optimal hydrochar-bacteria dosage for efficient contaminant removal and to avoid the unaffordable cost by the usage of large quantity of hydrochar.
4. A deeper understanding of activation material and activation process can improve the properties and enhance remediation efficiency.
5. Immobilizing genetically modified bacteria can reduce their performance limitations under harsh environmental conditions.
6. Field trials of hydrochar-bacteria integrated systems are required to validate their effectiveness and enable their practical adoption by the community.
7. Future research should focus on evaluating the regeneration, reusability, and long-term stability of hydrochar-bacteria composites under repeated adsorption-desorption cycles to determine their economic feasibility and suitability for large-scale environmental remediation.
